# Arachnoid web-a rare but surgically effectively treatable cause of spinal cord compression and syringomyelia

**DOI:** 10.1016/j.bas.2025.104336

**Published:** 2025-07-24

**Authors:** Chuh-Hyoun Na, Hani Ridwan, Georg Neuloh, Gerrit Alexander Schubert, Kay Nolte, Andreas Prescher, Hans Clusmann, Christian Blume

**Affiliations:** a Department of Neurosurgery, RWTH Aachen University Hospital, Germany; b Clinic for Diagnostic and Interventional Neuroradiology, RWTH Aachen University Hospital, Germany; c Institute of Neuropathology, RWTH Aachen University Hospital, Germany; d Institute of Molecular and Cellular Anatomy (MOCA), University RWTH-Aachen, Germany; e Department of Neurosurgery, Kantonsspital Aarau, Aarau, Switzerland

**Keywords:** Thoracic myelopathy, Dorsal arachnoid web, Scalpel sign, Spine surgery, Syringomyelia, CSF flow obstruction

## Abstract

**Objective:**

Dorsal arachnoid web (AW) is a rare cause of spinal cord compression, which is indicated on MRI by a dorsal indentation of the spinal cord (scalpel-sign). AW can be associated with a myelon lesion as a sign of secondary syringomyelia resulting from alterations of CSF flow-dynamics. The etiology of AW is unclear, and evidence-based treatment recommendations are still lacking.

**Methods:**

A retrospective chart study was conducted on patients with the scalpel-sign, treated at a tertiary neurospine center between 2016 and 2024. Clinical presentation, imaging, and histopathological findings were evaluated, and treatment outcome was assessed using the thoracic Japanese Orthopedic Association (tJOA) score pre- and postoperatively, and anatomically and pathophysiologically contributing factors are discussed.

**Results:**

17 patients (mean age 55.5 ± 10.3 yrs, 9 males) were identified. Predilection site was the upper half of the thoracic spine, with additional syringomyelia in 9 patients (53 %). 10 patients (65 %) showed sensory deficits, 10 (59 %) motor symptoms, 10 (59 %) ataxia, 9 (53 %) back pain, and 7 (41 %) neuropathic pain. 10 (59 %) patients underwent surgery with web removal/adhesiolysis. Median follow-up was 176 days. Postoperatively, symptoms worsened in one patient, but improved in the majority of cases (mean tJOA pre-/postoperatively: 8 ± 1.1/9.2 ± 1.3; Wilcoxon signed-rank-test p < .02), with postoperative MRI showing regression of AW and syringomyelia.

**Conclusion:**

Surgical intervention appeared beneficial in the majority of patients, even in those with longstanding symptoms and resulting in regression of spinal cord lesions. Awareness of AW should be raised, as it identifies a rare but effectively treatable cause of spinal cord compression and syringomyelia.

## Highlights

• Arachnoid web (AW) is a rare but potentially underdiagnosed cause of myelopathy.
• We analyzed clinical and imaging characteristics of AW and postoperative outcome.
• AW affected only the upper thoracic spine for which anatomical reasons are analyzed.
• Most AW patients improved postoperatively and showed regression of myelon lesions.

## 1 Introduction

Since the first anecdotal observations of a dorsal spinal arachnoid web (AW) as a potential cause of spinal cord compression and syringomyelia (Mallucci et al., 1997; Paramore, 2000), the awareness of this pathology and the number of cases reported in the literature have steadily increased since, especially after Reardon et al. (AJNR, 2013) characterized the ‘scalpel sign’ on MRI as a pathognomonic imaging feature, indicated by a dorsal indentation of the spinal cord. Up to now, however, the number of cases in the literature is limited, with only a fraction of those having been treated surgically. Therefore, clinical experience and evidence-based treatment recommendations are still extremely limited.

Intraoperatively, dorsal AW has been described as a firm arachnoidal band-like structure constricting the spinal cord at its dorsal aspect, with consecutive engorgement of the spinal cord. Frequently, an associated spinal cord lesion can be observed on MRI, which has been identified as syringomyelia secondary to spinal cord compression caused by alterations of CSF flow dynamics (Mallucci et al., 1997; Mauer et al., 2008; Mittal et al., 2024; Chang et al., 2014), but prevalence and clinical correlates thereof have remained somehow ill-defined. Previous reports suggest AW to be either a developing arachnoidal cyst or a remnant thereof, with both having been suggested to represent pathologies on a continuous spectrum rather than different entities (Chellathurai et al., 2018). While in some cases, preceding trauma, bleeding, or inflammatory conditions have been described as causative, the underlying etiology of AW remains elusive in the majority of cases. Notably, AW has in those ‘idiopathic’ cases consistently been reported to almost exclusively affect the thoracic myelon (Ben Ali et al., 2018; Bugdadi et al., 2023; Carr et al., 2024; Delgardo et al., 2022; Elkadi et al., 2023; Laxpati et al., 2021; Nisson et al., 2019), for which the reasons have not yet been identified.

Patients may present with progressive signs and symptoms of myelopathy, but the natural history of AW is unclear, and the criteria for triggering surgical intervention have yet to be determined.

We report a single tertiary neurospine center experience diagnosing and treating patients with AW. Clinical signs and symptoms, patients' demographics, imaging data and histopathological findings, as well as the postoperative outcome were evaluated and contextualized within the currently available literature. In addition, anatomically potentially predisposing factors and pathophysiological aspects contributing to syringomyelia formation are discussed.

## 2 Methods

Institutional review board approval was obtained (CTC-A 23–176, EK23-168) prior to initiation of the study, and patient consent was waived due to the retrospective nature of the analysis. A retrospective chart review was conducted on patients treated between 2016 and 2024 at a tertiary neurospine center who showed a scalpel sign on MRI. Clinical signs and symptoms, imaging findings, treatment outcome and –if available- results of histopathological analyses were evaluated. Treatment outcome was assessed pre- and postoperatively (t0, t1) using the thoracic Japanese Orthopedic Association (tJOA) score, which is used for clinical assessment of thoracic myelopathy (Aizawa et al., 2007; Palumbo et al., 2001) and comprises a maximum of 11 points, indicating more clinical impairment with lower scores. tJOA scores were also defined in the non-surgically treated patients at the time of diagnosis (t0) and at the time of last follow-up (t1). The Wilcoxon signed-rank-test, a non-parametric rank test, was applied to test for longitudinal tJOA changes with a significance level of p < .05.

## 3 Results

### 3.1 Patients' characteristics

**Fig. 1:**
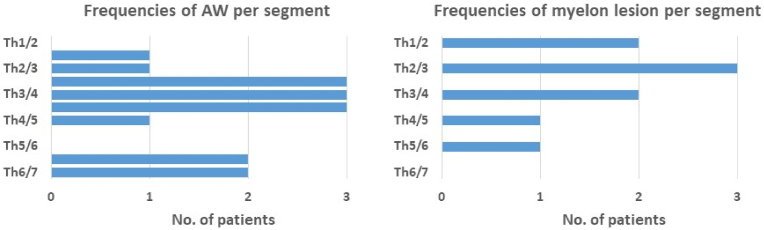
On the left: number of arachnoid webs (AW) per segment affected. Th: thoracic level. On the right: number of myelon lesions per segment. Total number of patients n = 17 with AW, of which n = 9 had an associated myelon lesion.

17 patients (9 male, mean age 55.5 ± 10.3 years) with typical scalpel sign on MRI were identified. In all patients, the scalpel sign was found at the mid to upper thoracic spine, with the maximal ventral displacement of the myelon found mostly at Th3-4. 9/17 (53 %) cases showed an associated centromedullary spinal cord lesion complying with syringomyelia, mostly at or cranially to the arachnoid web (see Fig. 1). Mean latency between clinical manifestation till diagnosing AW was 12.5 ± 10 months. Patients most commonly presented with sensory deficits (11/17, 65 %), motor symptoms of the legs (10/17, 59 %), ataxia (10/17, 59 %), diffuse back pain (9/17, 53 %) and neuropathic pain (7/17, 41 %). Bladder dysfunction was encountered in only one patient, who however had previously received prostate cancer treatment (for patients' demographic and clinical details please see Table 1).

**Table 1:** Demographic and clinical data of patients.

Pt.	Age	Gender	AW	ML	Mot.	Sens.	NP	Ataxia	Bladder	Back pain	Surgery	Follow-up	tJOA	tJOA
					t0/t1	t0/t1	t0/t1	t0/t1	t0/t1	t0/t1	Y/N	(days)	t0	t1
1	49	f	th4/5	–	+/+∗	+/+∗	+/−	+/+∗	−/−	−/−	Y	4	7	8
2	57	f	th6/7	th5/6	+/−	+/+	−/−	+/+∗	−/−	−/−	Y	641	8	9
3	74	f	th3	th2/3	−/−	−/−	−/−	+/−	−/−	−/−	Y	178	9	11
4	53	f	th5/6	th5	+/−	+/+	−/−	+/+	−/−	−/−	Y	125	7	9
5	44	m	th3	th2/3	+/+	+/+	+/−	+/+∗	−/−	−/−	Y	4	7	8
6	67	m	th3	–	−/−	−/−	+/+	−/−	−/−	+/+	N	683	11	11
7	40	m	th4	th3/4	+/+	+/+	+/+	−/−	−/−	+/+	N	749	8	8
8	57	m	th4	th3/4	−/−	+/+	+/+	+/+	−/−	+/+	N	261	9	9
9	56	m	th3/4	th2/3	+/−	−/−	−/−	+/−	−/−	+/−	Y	3	10	11
10	75	m	th2	th1/2	−/−	+/+	−/−	−/−	+/+^#^	+/+	Y	173	8	8
11	50	f	th6/7	–	+/−	+/−	−/−	−/−	−/−	+/−	Y	116	7	11
12	51	f	th6	–	−/−	−/−	−/−	−/−	−/−	+/+	N	19	11	11
13	58	f	th3/4	–	+/+	+/+	−/−	−/−	−/−	+/+	N	695	9	9
14	61	m	th4	–	+/+∗∗	+/+	−/−	+/+∗∗	−/−	−/−	N	1232	9	6^##^
15	39	m	th3/4	th1/2–4/5	+/−	−/+	+/+	−/+	−/−	−/−	Y	721	9	8^###^
16	62	m	th6	–	−/−	+/+	+/+	+/−	−/−	−/−	Y	94	8	9
17	60	m	th2/3	th2	−/−	−/−	−/−	+/+	−/−	+/+	N	196	10	10

**AW:** level of arachnoid web; **ML**: myelon lesion; **Mot.:** motor symptoms; **Sens.:** sensory symptoms; **NP:** neuropathic pain; **Bladder:** bladder dysfunction; **t0:** time of diagnosis; **t1:** time of last follow-up; **Y/N: yes/no; +/−:** present/absent; **tJOA:** thoracic Japanese Orthopedic Association score; **th:** thoracic level; **∗:** relatively improved;**∗∗:** worsened; **#:** history of prostate cancer; **##:** history of encephalitis disseminata; **###:** early postoperative hemorrhage with secondary myelon adhesions.

### 3.2 Surgical intervention

**Fig. 2:**
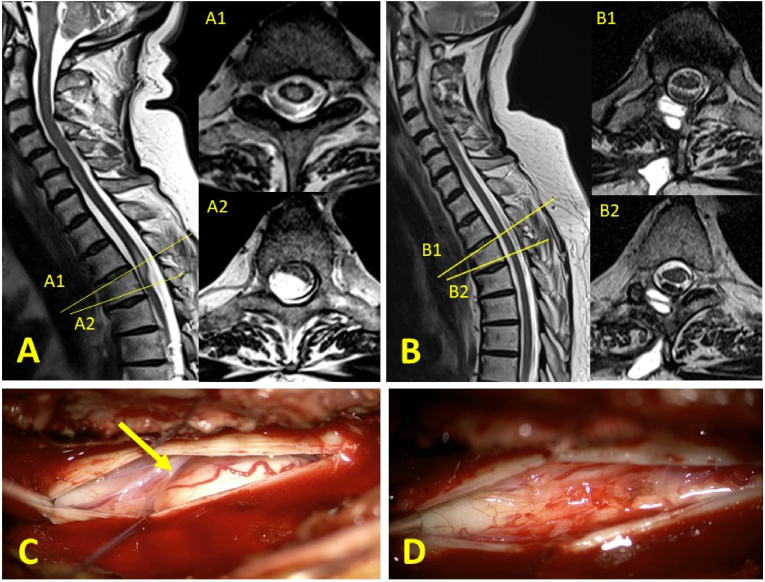
A: preoperative MRI of a patient with classical ‘scalpel sign’ on high-resolution T2-weighted MR imaging of the spinal cord. A1: axial plane at the segment of spinal cord lesion, A2: at the segment of the AW with maximal ventral myelon displacement/dorsal indentation. B: postoperative MRI of the same patient showing resolution of ventral myelon displacement and myelopathy, corresponding to postoperative clinical recovery. C: intraoperative view on the AW. D: after web removal.

10 patients (with progressive clinical symptoms and/or progressive myelon lesion) were surgically treated by mono- or bisegmental hemilaminectomy, durotomy with web dissection/adhesiolysis, and dural closure. Intraoperatively, arachnoid webs presented as firm band-like subarachnoidal structures traversing the dorsal aspect of the myelon obliquely in the craniocaudal direction (see Fig. 2). In four patients, motor and sensory evoked potentials were monitored intraoperatively.

### 3.3 Histopathological findings

**Fig. 3:**
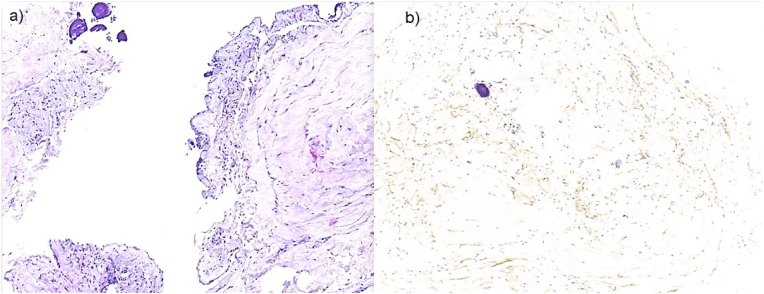
Tissue specimen analyzed after web-removal. Histopathological examination revealed a loose meshwork of connective tissue, focally enriched in collagen fibers and harbouring intermingled EMA-positive meningothelial lamellae. Sometimes, meningo-/arachnothelial cells cover the web. Occasionally, psammoma bodies (upper left in a) and b)) are found, also indicating involvement of meningothelial structures. No signs of arachnoiditis or former bleeding were encountered. a) H&E, b) Immunohistochemical stain for epithelial membrane antigen (EMA).

Histopathological analysis was performed in 7 out of 10 surgically treated patients. The specimen typically showed collagen-enriched mesenchymal tissue with intermingled bands of arachnoid cells. Occasionally, psammoma bodies were encountered indicating involvement of meningo-/arachnothelial structures. Correspondingly, on immunohistochemistry fine EMA- (Epithelial membrane antigen) positive lamellae could be visualized. In none of the cases, signs of inflammation, remnants of significant bleeding, or hints of neoplasia were found (see Fig. 3). While a previous study reported in 26 % of analyzed specimens signs of calcification (Carr et al., 2024) which authors suggested as potential indicator of arachnoiditis ossificans, no such changes were observed in the present cohort.

### 3.4 Treatment outcome

**Fig. 4:**
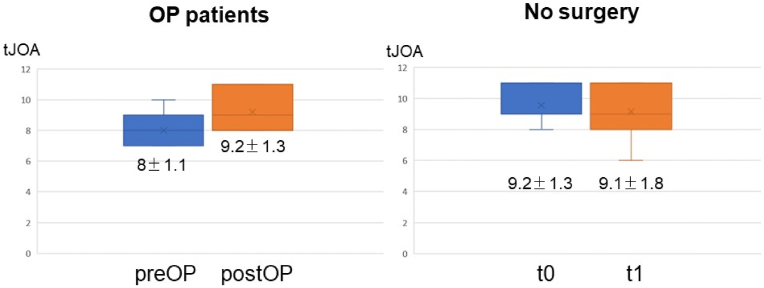
Graphs show mean values of tJOA scores with standard deviations for the surgically treated patient group pre- and postoperatively and for patients without surgery the time of diagnosis (t0) and at the time of latest follow-up (t1).

Out of 10 surgically treated patients, the majority of cases (8/10) improved postoperatively, while in one patient, symptoms remained unchanged. One patient, however, who suffered early postoperatively minor sub-/epidural hemorrhage deteriorated clinically, with secondary myelon adhesions and syringomyelia formation found on MRI follow-up. However, in all other surgically treated patients who received postoperative MRI (8/10), spinal cord lesions and the scalpel sign completely resolved postoperatively (see Fig. 2). With a median follow-up of 176 days, mean tJOA (preoperatively 8 ± 1.1) improved significantly after surgery (9.2 ± 1.3) in the surgically treated patient group (Wilcoxon signed-rank-test p < .02), while no change was observed in non-surgically treated patients (tJOA t0: 9.2 ± 1.3; tJOA t1: 9.1 ± 1.8) (see Fig. 4).

## 4 Discussion

We present a single center experience treating a series of patients with arachnoid web (AW) as identified by the scalpel sign on spinal MRI. Most patients presented with back pain and varying degrees of thoracic myelopathy symptoms. We observed the scalpel sign exclusively in the mid to upper thoracic spine, which is consistent with previous observations reported in the literature (Ben Ali et al., 2018; Carr et al., 2024; Delgardo et al., 2022; Elkadi et al., 2023; Laxpati et al., 2021; Nisson et al., 2019). About half of our patients (53 %) showed an associated centromedullary spinal cord lesion compatible with syringomyelia which was found either at, or just cranial to the level of maximal dorsal myelon indentation.

Differential diagnoses upon referral to our clinic comprised ‘intramedullary tumor’, ‘myelomalacia of unknown origin’, ‘myelitis’, ‘ventral myelon adherence of unknown origin’, ‘intraspinal arachnoidal cyst’ or ‘ventral myelon herniation’. Such diagnostic uncertainty and the long mean latency (12.5 months) between beginning of clinical manifestation and diagnosing AW suggests, that AW might still often remain underdiagnosed, but with increasing awareness of this condition being noticeable in recent years.

Complying with the known literature, our patients had no preceding trauma, bleeding, nor inflammatory condition evident in the past medical history, which is consistent with our histopathological analyses, showing no signs of arachnoiditis. While the etiology of AW remains unclear, the thoracic spine seems to offer anatomically predisposing factors which we discuss in the following.

### 4.1 Anatomic features of the thoracic subarachnoid compartment

**Fig. 5:**
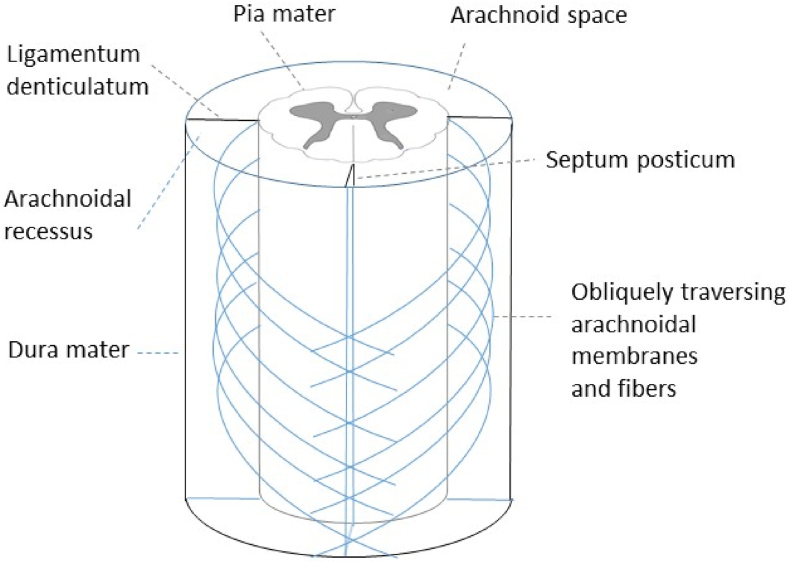
Schematic view onto the dorsal aspect of the thoracic subarachnoidal space. (Adapted from: Praktische Anatomie (v. Lanz/Wachsmuth) Band 2/7 (Rücken) Springer 1982).

Within the spinal canal, the spinal cord is suspended by multiple reticular and elastic fibers and membranes, connecting the lamina externa of the pia mater spinalis to the dura mater, and thereby preventing the spinal cord of major displacement, as well as buffering traumatic impact. These fibers originate from the lamina externa (epipia) and follow craniocaudally an oblique, partially longitudinal direction, blending into surrounding structures and causing a grate-like net structure (Rickenbacher et al., 1982). Originating from the lamina externa and spanning (from the cervical to the upper lumbar spine) to the dura mater in a coronal plane is the ligamentum denticulatum, which divides the subarachnoid space into a ventral and dorsal compartment. Dorsally to the ligamentum denticulatum, multiple membranes and trabeculae connect the arachnoida to the pia mater, most densely in the midsagittal dorsal plane, forming the septum posticum, whereby the dorsal subarachnoid space is further subdivided into a left and right compartment (please see Fig. 5). The septum posticum can be splintered into multiple lamellae forming membraneous subcompartments, with multiple connective tissue trabeculae interconnecting dorsal spinal nerve roots laterally to the ligamentum denticulatum and the arachnoidea. Along the thoracic spine, those trabeculae form fenestrated membranes, crossing obliquely towards the next caudal segment, thereby forming subcompartments (recessus laterales obliqui) between neighbouring spinal cord roots (Key and Retzius, 1875).

Consequently, CSF flow at the thoracic level is dorsally more obstructed than ventrally. CSF flow-velocities on the other hand have been observed to be the highest within the thoracic spinal canal (Freund et al., 2001), which is smaller in diameter as compared to the cervical or the lumbar spinal canal. Moreover, during ventral flexion, the thoracic dorsal spinal cord plane has been found to extend up to 6 cm in the craniocaudal direction with ventral displacement of the myelon (Breig, 1960).

In our patients, the AWs encountered intraoperatively traversed the dorsal aspect of the spinal cord obliquely in craniocaudal direction (see Fig. 2 C), which corresponds to the obliquely traversing subarachnoidal fiber- and membraneous structures described above. Given the dorsally more densely, grate-like structured subarachnoidal compartment, the locally increased CSF flow-dynamics, and the higher biomechanical strain on the kyphotic spinal cord during flexion may thus in sum predispose to AW formation preferentially at the upper-to-mid thoracic level.

### 4.2 Pathophysiological mechanisms of syringomyelia formation

**Fig. 6:**
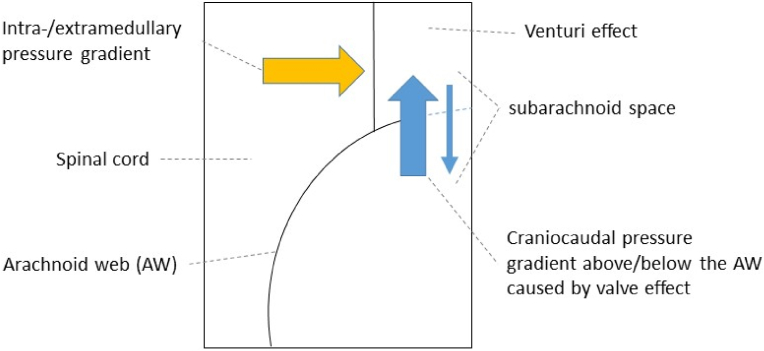
Schematic view onto the spinal canal in the sagittal plane with pathophysiological factors driving syringomyelia formation in patients with arachnoid web (AW). **Yellow arrow**: Intra-/extramedullary pressure gradient caused by flow obstruction within the canalis centralis with intramedullary accumulation of CSF. **Blue arrow**: craniocaudal pressure gradient above/below the AW caused by craniocaudally restricted CSF flow within the subarachnoid space with valve effect of the AW allowing CSF flow in one direction, but less so in the opposite direction, leading to entrapment of CSF on one side of the AW with increasing pressure onto the spinal cord and pressure gradient within the subarachnoid compartment. **Venturi-effect**: caused by narrowing of the subarachnoid space with ensuing increase of local CSF flow velocity, which is assumed to exert a suction effect on the myelon.

While the pathophysiology for syringomyelia formation associated with AW is not fully understood, several contributing factors have been suggested in the literature. Craniocaudal CSF flow obstruction caused by AW has previously been suggested to effect a locally increased pulse pressure within the subarachnoid space, forcing CSF into the myelon (Williams,1980). Greitz (2006) on the contrary rather suggested an increased intramedullary pulse pressure relative to the subarachnoid space as the distending force, leading to syringomyelia formation by intramedullary accumulation of extracellular fluid. He further introduced the ‘Venturi Effect’ as a contributing pathophysiological factor, describing an increase in flow velocity to be accompanied by a decrease of pressure of fluids passing through narrowed pathways, which was suggested to exert a suction effect on the myelon by pressure decrease within the narrowed subarachnoid compartment of AW patients. In our cohort, we observed syringomyelia at or more commonly above the level of myelon compression. This corresponds to findings by Chang et al. (2014), who suggested a one-way valve like effect caused by the AW, which allows pulsed CSF flow craniocaudally into one, but less so in the opposite direction, leading to entrapment of CSF on one side of the AW, and thereby causing a pressure gradient above and below the AW (see Fig. 6). Thus, syringomyelia formation may well be caused by a combination of a craniocaudal, but as well intra-/extramedullary pressure gradient caused by the AW, which is consistent with previous observations (Mittal et al., 2024; Sridharan and Heilman, 2009; Chang et al., 2014).

### 4.3 Diagnostics

**Fig. 7:**
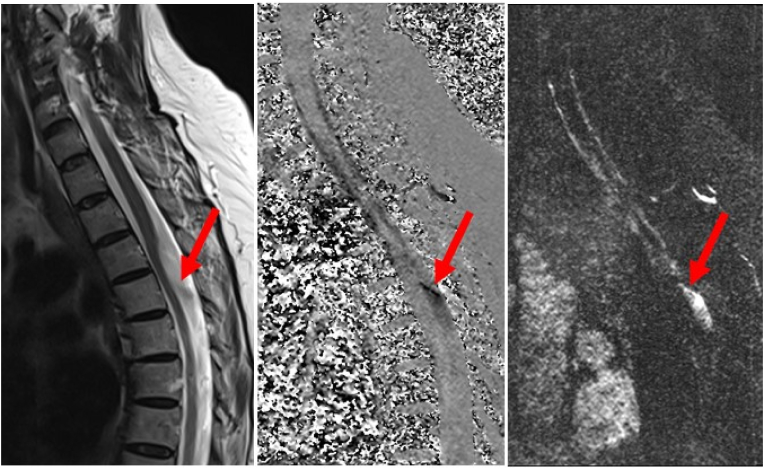
Using cardiac-gated phase-contrast cine-mode MRI, CSF pulsations were found to be interrupted during systole and diastole below the dorsal indentation of the spinal cord, thereby delineating the obstruction of CSF flow caused by the AW.

While contrast-enhanced MRI and CT-myelography had been additionally applied in some of the patients to exclude inflammatory or neoplastic conditions or myelon herniation, these imaging modalities do not seem mandatory for diagnosing AW. In those patients with a typical scalpel sign, high-resolution T2-weighted MRI sufficed to non-invasively diagnose AW in our patient group. Advanced MR-imaging techniques as such as heavily T2-weighted constructive interference in steady state (CISS) sequences or cardiac-gated phase-contrast cine-mode MRI of CSF (Mauer et al., 2008; Chang et al., 2014; Grewal et al., 2015) may further increase diagnostic certainty by delineating CSF flow obstructions, especially in those cases where myelon compression might only be subtle and syringomyelia absent. Performing quantitative CSF flow-measurements perpendicular to the axial plane at different levels above and below the site of spinal cord compression, Chang et al. (2014) demonstrated a ball-valve effect with restricted craniocaudal CSF pulsations due to AW. Accordingly, applying cardiac-gated phase-contrast cine-mode MRI in one of our patients, CSF pulsations were found to be interrupted during systole and diastole below the dorsal indentation of the spinal cord (please see Fig. 7), allowing to delineate the obstruction of CSF flow caused by AW.

Correspondingly, Mauer et al., (2008) previously identified in 33 out of 125 patients with syringomyelia of unknown origin otherwise inapparent CSF flow-obstructions by analyzing CSF flow dynamics using cardiac-gated phase-contrast cine-MRI. In 8 out of these 33 patients, additional CT-myelography was performed, but showed CSF flow obstruction only in 2 of these 8 cases. Therefore, CT-myelography was suggested to not be the optimal imaging modality for diagnosing AW, as it appears to be more sensitive to restrictions of low-flow or complete obstructions, rather than capturing alterations in CSF pulsations. CT–myelography might however be helpful in differentiating AW from arachnoidal cysts, which are delineated by delayed filling compared to the surrounding subarachnoid space (Reardon et al., 2013). In patients with typical scalpel sign at the upper to mid thoracic level on MRI however, CT-myelography seems to be dispensable considering the limited additional information and the invasive nature with inherent risks of secondary complications (Sandow and Donnal, 2005).

Some authors report the use of intraoperatively applied dye (gentian violet) (Yamaguchi et al., 2015), or intraoperative ultrasound for localizing the site of CSF flow obstruction, as well as to confirm restoration of CSF flow after web removal (Voglis et al., 2022; Delgardo et al., 2022; Laxpati et al., 2021; Castillo et al., 2023).

### 4.4 Therapeutic intervention in AW

Different surgical approaches have been reported in the past, with surgical intervention having been found to result in a beneficial outcome in the majority (91 %) of cases (Nisson et al., 2019), although this depends on the type of surgical intervention chosen. Early treatment reports included sporadically described web divisions with or without insertion of a stent (Mallucci et al., 1997; Brodbelt and Stoodley, 2003), myelotomy for release of syringomyelia with unreported outcome (Reardon et al., 2013), and singular cases of syringopleural shunting, with the latter however resulting in clinical deterioration and even paraplegia in one of the reported patients (Mallucci et al. 1997). More recently, a few cases treated using percutaneous fenestration of the web by introducing a lumbar catheter into the spinal canal have been reported with a favorable outcome (Qureshi et al., 2020; Nada et al., 2020).

However, laminectomy or hemilaminectomy with durotomy and web dissection has emerged as by far the most commonly reported procedure (Delgardo et al., 2022; Elkadi et al., 2023; Hirai et al., 2018; Mallucci et al., 1997; Paramore, 2000; Nisson J et al., 2019; Chang et al., 2014; Grewal et al. 2015; Yamaguchi et al., 2015; Laxpati et al., 2021; Bugdadi et al., 2023; Carr et al., 2024).

In view of the pathophysiological sequelae of CSF flow obstructions caused by AW, surgical intervention thus has to primarily aim at a restoration of normal CSF flow dynamics by removal of the flow obstruction (rather than at e.g. surgical drainage of syringomyelia), and has to prevent secondary arachnoiditis and adhesions following surgical intervention.

Based on our single center experience, we therefore advocate for surgical intervention as minimally invasive as possible (however taking into account the obliquely traversing fiber structure of the AW, which is most likely to craniocaudally exceed the segment of maximal dorsal myelon indentation delineated on MRI), with careful microsurgical dissection of the AW and, if attainable without severe manipulation, removal of the web, but without excessive attempts of lysis, using thorough saline irrigation and meticulous hemostasis preceding dural closure in order to minimize the risk of secondary arachnoiditis and adhesions. Regarding the single case in our study who suffered worsening of symptoms after early postsurgical hemorrhage with ensuing secondary adhesions and myelopathy on follow-up MRI, it has to be noted that this was the only case in which the myelon lesion extended preoperatively over three segments (Th1/2-Th4/5). Therefore, in this particular case, surgical intervention (with hemilaminectomy at Th3, Th4, and partial hemilaminectomy at Th5, with lateral and ventral exploration and difficult hemostasis intraoperatively requiring application of tabotamb) seemed to have caused more manipulation and trauma than in the other cases treated, thereby increasing the risk of secondary hemorrhage, arachnoiditis and myelon adhesions. Moreover, AW patients have to be carefully identified and distinguished from those cases with secondary arachnoiditis or syringomyelia due to other causes, as the required surgical strategy and the operative outcome will substantially differ depending on the underlying pathology. At present, only those patients with significant or progressive symptoms are treated surgically in our clinic, while we aim to further monitor also non-surgically treated patients, as the natural history and disease dynamics of AW are unknown.

### 4.5 Limitations

As we obtained retrospective data from only a small series of patients with varying follow-up intervals, generalizability of our findings is certainly limited, and a long-term beneficial effect of surgical intervention cannot be validated yet. Comparison of surgically and non-surgically treated patients was not feasible in the present study, as non-surgically treated patients were less severely affected and showed overall higher tJOA scores at the time of diagnosis. Potential confounders can furthermore be introduced by comorbidities (e.g. with one of our patients having a history of prostate cancer, and one case with a history of encephalitis disseminata), which may cause etiological attribution of clinical symptoms to be equivocal. Moreover, inter-rater reliability regarding tJOA assessment was not evaluated in the current study. Although the mJOA (from which the tJOA has been derived) has previously been described with a high intraclass correlation coefficient of 0.88 [Martin et al., 2021], it has to be considered that interrater variability introduces variance and potential inaccuracies in the clinical assessment of patients, which seems crucial especially with regard to longitudinal assessments and evaluation of treatment outcome. Therefore, future prospective studies should address this aspect by including clinical assessment by at least two experienced clinicians, rating patients independently in order to be able to estimate and to correct for interrater-variability in the analysis. Randomized prospectively acquired multicentric patient data are necessary to gain a better understanding of the natural history and disease dynamics, and to thereby obtain further guidance in treatment decisions. Despite these limitations, our study not only adds to the known literature, but provides a comprehensive view on clinical and diagnostic features of this rare condition, and contributes new aspects by discussing anatomically potentially predisposing factors, accounting for the thoracic spine as a predilection site of AW formation.

## 5 Conclusion

Surgical intervention appeared beneficial in the majority of AW patients, even in those with longstanding symptoms and resulting in regression of spinal cord lesions. Patients should be carefully screened for AW, as it identifies a rare but surgically effectively treatable cause of spinal cord compression and syringomyelia. Increasing awareness of this rare condition and obtaining a better understanding of the underlying pathomechanisms may aid to improve patient care and to economize diagnostic procedures. Prospective longitudinal studies of both, surgically and non-surgically, treated AW patients should be conducted in order to gain a better understanding of the natural history of the disease, and to obtain further guidance in treatment decisions.

## Authorship

Conception and design of the study: CN Acquisition of data: CN, HR, GN, GS; Analysis and interpretation of data: CN, HR, KN; Drafting the article: CN; Revising the article: HC, CB, AP, HR, GN, KN, GS; Final approval of the version to be submitted: CN, HR, AP, CB, HC, GN, KN, GS.

## Funding

This research project was not funded.

## Declaration of competing interest

The authors declare that they have no known competing financial interests or personal relationships that could have appeared to influence the work reported in this paper.
